# Insight into the Mechanism of Interactions between the LL-37 Peptide and Model Membranes of *Legionella gormanii* Bacteria

**DOI:** 10.3390/ijms241512039

**Published:** 2023-07-27

**Authors:** Katarzyna Pastuszak, Bozena Kowalczyk, Jacek Tarasiuk, Rafal Luchowski, Wieslaw I. Gruszecki, Małgorzata Jurak, Marta Palusinska-Szysz

**Affiliations:** 1 Department of Interfacial Phenomena, Institute of Chemical Sciences, Faculty of Chemistry, Maria Curie-Skłodowska University, Maria Curie-Skłodowska Sq. 3, 20-031 Lublin, Poland; katarzyna.pastuszak@mail.umcs.pl; 2 Department of Genetics and Microbiology, Institute of Biological Sciences, Faculty of Biology and Biotechnology, Maria Curie-Skłodowska University, Akademicka 19, 20-033 Lublin, Poland; b.kowalczyk746@wp.pl (B.K.); jacek.tarasiuk@mail.umcs.pl (J.T.); marta.palusinska-szysz@mail.umcs.pl (M.P.-S.); 3 Department of Biophysics, Institute of Physics, Faculty of Mathematics, Physics and Informatics, Maria Curie-Skłodowska University, Radziszewskiego 10, 20-031 Lublin, Poland; rafal.luchowski@mail.umcs.pl (R.L.); wieslaw.gruszecki@mail.umcs.pl (W.I.G.)

**Keywords:** *Legionella gormanii*, LL-37, Langmuir monolayer technique

## Abstract

*Legionella gormanii* is a fastidious, Gram-negative bacterium known to be the etiological agent of atypical community-acquired pneumonia. The human cathelicidin LL-37 exhibits a dose-dependent bactericidal effect on *L. gormanii*. The LL-37 peptide at the concentration of 10 µM causes the bacteria to become viable but not cultured. The antibacterial activity of the peptide is attributed to its effective binding to the bacterial membrane, as demonstrated by the fluorescence lifetime imaging microscopy. In this study, to mimic the *L. gormanii* membranes and their response to the antimicrobial peptide, Langmuir monolayers were used with the addition of the LL-37 peptide to the subphase of the Langmuir trough to represent the extracellular fluid. The properties of the model membranes (Langmuir monolayers) formed by phospholipids (PL) isolated from the *L. gormanii* bacteria cultured on the non-supplemented (PL−choline) and choline-supplemented (PL+choline) medium were determined, along with the effect of the LL-37 peptide on the intermolecular interactions, packing, and ordering under the monolayer compression. Penetration tests at the constant surface pressure were carried out to investigate the mechanism of the LL-37 peptide action on the model membranes. The peptide binds to the anionic bacterial membranes preferentially, due to its positive charge. Upon binding, the LL-37 peptide can penetrate into the hydrophobic tails of phospholipids, destabilizing membrane integrity. The above process can entail membrane disruption and ultimately cell death. The ability to evoke such a great membrane destabilization is dependent on the share of electrostatic, hydrogen bonding and Lifshitz–van der Waals LL-37−PL interactions. Thus, the LL-37 peptide action depends on the changes in the lipid membrane composition caused by the utilization of exogenous choline by the *L. gormanii*.

## 1. Introduction

*Legionella* spp. are ubiquitous Gram-negative bacteria that can cause a range of diseases, including self-limited influenza-like syndrome (Pontiac fever) and pneumonia. Pneumonia is the most common clinical manifestation of Legionnaires’ disease. *L. pneumophila* is widely recognized as a primary causative agent of both community-acquired and nosocomial pneumonia. The clinically relevant non-pneumophila species include *L. longbeachae*, *L. micdadei*, *L. bozemanae*, *L. dumoffii*, and *L. gormanii*. *L. gormanii* primarily affects children and immunodeficient patients [1,2]. However, immunocompetent individuals can also be susceptible to infection by this pathogen [3]. The pathogenicity of *Legionella* bacteria, which involves numerous virulence factors, is attributed to their capacity to proliferate within human macrophages, evading their bactericidal mechanisms. Genetic analysis has proven that *L. gormanii* encodes approximately 130 putative virulence factors from 300–400 putative virulence genes present in *L. pneumophila* [4]. Among these virulence factors, *L. gormanii* harbors genes that encode homologs of diverse secretion systems, including the Dot/Icm (defective in organelle trafficking/intracellular multiplication) type IV secretion system [5]. This multiprotein system, involved in establishing an intracellular replication niche (LCV-*Legionella* containing vacuole) by translocating macromolecules across the host cell membranes, consists in the effective functioning of the bacterial cell envelope.

The cell envelope of Gram-negative bacteria consists of two membranes: the outer membrane and the cytoplasmic membrane. The cytoplasmic membrane is composed of a phospholipid bilayer, whereas the outer membrane is made up of an inner leaflet of phospholipids and an outer leaflet of lipopolysaccharide (LPS). The lipidomic analysis of *L. gormanii*’s outer (OM) and inner (IM) membranes using liquid chromatography and mass spectrometry revealed that the bacteria synthesized a wide range of lipids. The most abundant lipid fraction in both layers were phospholipids (phosphatidylcholine, PC; phosphatidylethanolamine, PE; phosphatidylglycerol, PG; cardiolipin, CL), followed by glycerolipids (triglyceride, TG; diglyceride, DG) and sphingolipids (ceramides, Cer; hexosylceramides, Hex1Cer) [6]. The distinctive feature of *L. gormanii* is its ability to synthesize PC and ceramides, the characteristic constituents of eukaryotic membranes. *L. gormanii* can produce PC via two independent pathways, i.e., the phospholipid N-methylation (PMT) pathway and the phosphatidyl-choline synthase (PCS) pathway. In the PMT pathway, PE is subjected to a series of methylation reactions catalyzed by the phospholipid *N*-methyltransferase encoded by the *pmtA* gene. However, *L. gormanii* lacks the *pmtA* gene homologous to other *Legionella*, *Rhodobacter*, and *Rhizobium* bacteria. The second pathway is promoted by the PcsA enzyme, which condenses exogenous choline and CDP-diacylglyceride (cytidine diphosphate) to produce PC in a one-step reaction. The *L. gormanii* PcsA protein is 254 amino acids long and highly hydrophobic, containing up to 8 transmembrane helices with the N- and C-termini inside the bacterial cell [7]. PcsA depends on the microorganism interactions with the eukaryotic hosts or access to the eukaryotic metabolites since choline is not a biosynthetic product of prokaryotes.

*L. gormanii* cultured on the choline-supplemented medium (PL+choline) demonstrates quantitative differences in the molecular profile of phospholipids compared to the bacteria cultured without the addition of exogenous choline (PL−choline) [6]. The lipids isolated from *L. gormanii* cultured on the medium without the addition of choline contained 26% PC, 50% PE, 21% CL, and 3% PG, while those grown on the medium with choline contained 47% PC, 38% PE, 12% CL, and 3% PG [7]. These differences determine the physicochemical properties of the membranes formed by lipids. The monolayers built of lipids isolated from *L. gormanii* cultured with the addition of exogenous choline were more ordered and densely packed at 20 °C and 37 °C compared to the monolayers formed from the bacteria cultured without the addition of choline, as indicated by the higher values of the compression modulus of these monolayers. The tighter packing of the molecules contributed to the increased ordering of the acyl chains. The higher degree of condensation of molecules in the monolayers formed from the *L. gormanii* lipids cultured on the medium supplemented with choline resulted from the favorable interactions mediated by the hydrogen bonds and Lifshitz–van der Waals forces between the polar groups of phospholipids and the long chains of C19–C21 fatty acids (FA), respectively. The Brewster angle microscope images revealed that the monolayers formed from the lipids isolated from *L. gormanii* cultured in the medium with exogenous choline were more homogeneous than those formed from the lipids of bacteria cultured without the addition of exogenous choline. The monolayers of lipids extracted from bacteria cultured on the medium without the addition of choline displayed increased flexibility attributed to the presence of shorter chains (C14–C18) of unsaturated fatty acids, as well as a larger content of cardiolipin (21%), leading to the formation of heterogeneities (domains) [8].

The utilization of exogenous choline by *L. gormanii* leads not only to alterations in the lipid profile but also affects proteins, enabling these bacteria to modulate the immune response to infection. When *L. gormanii* is cultured on the medium supplemented with choline, it induces the production of pro-inflammatory cytokines IL-6 and TNF-α to a lesser extent compared to the bacteria grown on the medium without exogenous choline [7].

To combat *Legionella* spp. in the lungs, cationic antimicrobial peptides like human β-defensin 3 (hBD-3) and the LL-37 peptide play a crucial role. Pulmonary cells produce hBD-3 during an *L. pneumophila* infection via the TLR-JNK-AP-1 (Toll-Like Receptor-c-Jun N-terminal Kinase-Activator Protein-1) pathway [9]. The LL-37 peptide, known as human cathelicidin, is expressed in various cell types, including epithelial cells, macrophages, neutrophils, and natural killer (NK) cells [10]. The LL-37 peptide exhibits antimicrobial activity by interacting with bacterial cell targets, including membrane lipids, thereby initiating and regulating the innate immune response [11]. Legionnaires’ disease caused by *L. gormanii* is a severe infection, particularly among immunocompromised patients. Making a rapid diagnosis and effective antibiotic treatment are crucial for reducing mortality rates. The LL-37 peptide, with its broad-spectrum activity including bactericidal effects and immune modulation potential, is considered a potential therapeutic alternative to conventional antibiotics [12].

The aim of this study is to demonstrate that the LL-37 peptide, depending on the applied concentration, exhibits either bactericidal activity against *L. gormanii* cells or induces their transition into a viable but non-culturing state. The ability of *L. gormanii* cells to interact with the LL-37 peptide is demonstrated by measuring the excitation energy transfer of fluorophore located on the surface of the bacteria and the labeled LL-37 peptide. Membrane penetration tests are carried out using the Langmuir monolayer technique to investigate the bactericidal mechanism of the LL-37 peptide on *L. gormanii* membranes. The lipids used to form the membranes are isolated from the *L. gormanii* cells cultured on the choline-supplemented and non-supplemented media, aiming at the investigation of how membrane composition modulates the interactions with this peptide.

## 2. Results

### 2.1. The LL-37 Peptide Exerts an Antibacterial Effect on L. gormanii Cells

**Figure 1:**
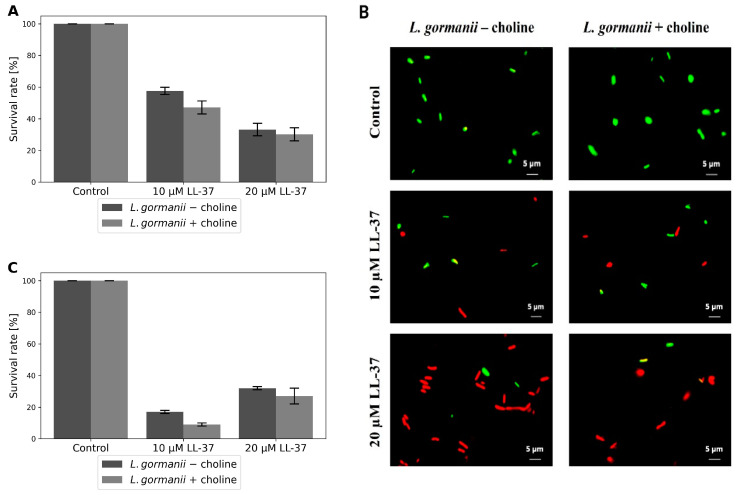
Effect of the LL-37 peptide on the survival rate (%) of *L. gormanii* cultured with and without choline supplementation using (**A**) live/dead staining and (**C**) colony-counting assay. (**B**) Live/dead staining *L. gormanii* cultured with and without choline supplementation after 1-h treatment with 10 µM and 20 µM LL-37 peptide, respectively, and imaged with laser scanning confocal microscopy (LSCM). The green signal is due to the dye SYTO9, indicating live cells, while the red signal is due to PI, which designates dead cells. Magnification 1000×.

The antibacterial activity of the LL-37 peptide on *L. gormanii* cells was assessed using double staining with SYTO9 and propidium iodide, enabling differentiation between live and dead cells based on membrane integrity. This peptide exerted a dose-dependent killing effect on *L. gormanii* cultured with and without choline supplementation (Figure 1A,B). At the concentration of 10 µM, the LL-37 peptide caused an approximate 40% decrease in the viability of *L. gormanii* cultured on the medium without the addition of choline, and an approximate 50% decrease in the viability of the bacteria grown on the medium with exogenous choline. The LL-37 peptide at a concentration of 20 µM further decreased cell viability, with a less pronounced difference observed between the bacteria cultured with and without extracellular choline.

The colony-counting plate method showed that the LL-37 peptide at the concentration of 20 µM resulted in a 68% decrease in cell viability of *L. gormanii* cells cultured on the medium without the addition of choline and a 72% decrease in cell viability grown on the medium supplemented with exogenous choline (Figure 1C). The survival rate of bacteria after treatment with the LL-37 peptide at a concentration of 20 µM was the same as that obtained in the live/dead staining test. However, after applying a 10 µM concentration of the LL-37 peptide, the rate of mortality was 83% and 91% for the bacteria cultured without and with the addition of choline, respectively, and was higher than that obtained in the live/dead staining method. The disparity in the bacterial survival rate between the two methods is likely due to the fact that the LL-37 peptide at the concentration of 10 µM, besides the killing effect, renders the bacteria viable but incapable of forming colonies on the BCYE medium (VBNC, viable but nonculturable).

The bacteria grown on the medium without and with exogenous choline after incubation with 10 µM of the LL-37 peptide and plated on the medium with the addition of 1% monosodium glutamate and 0.5% sodium pyruvate formed 17% and 23% more colonies, respectively, compared to those grown on the medium without these additives. These findings suggest that the viable but non-culturable forms of *L. gormanii* induced by the LL-37 peptide have been revived to the viable and cultivable state.

### 2.2. FLIM-FRET Measurements

**Figure 2:**
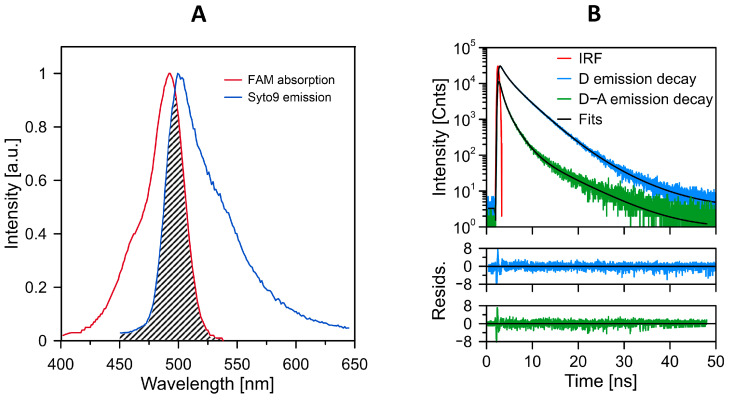
Spectroscopic and dynamic characterization of the pair of fluorophores FAM and Syto 9. (**A**) Fluorescence emission spectrum of excitation energy donor (Syto 9) and absorption spectrum of energy acceptor (FAM). The spectra were normalized at the maximum. The spectral overlap area is designated. (**B**) The original fluorescence decay traces and multiexponential fits of Syto 9 incorporated into the bacterial membrane (D) and the same system after the exposition to the LL-37 peptide labeled with FAM (D−A). The IRF trace is also shown. The residuals confirming the accuracy of the fits are shown in the lower panels.

The phenomenon of nonradiative excitation energy transfer was applied to examine the interactions of the LL-37 peptide with bacterial membranes. Both the protein and membranes were labeled with fluorophores that satisfy the conditions for an efficient excitation energy transfer, providing preferable orientation and close distance between the energy donor and the acceptor molecules: FAM labeled the LL-37 peptide, and Syto 9 labeled the membranes. The fluorescence labels for emission spectrum of the donor and the absorption spectrum of the acceptor are shown in Figure 2.

**Figure 3:**
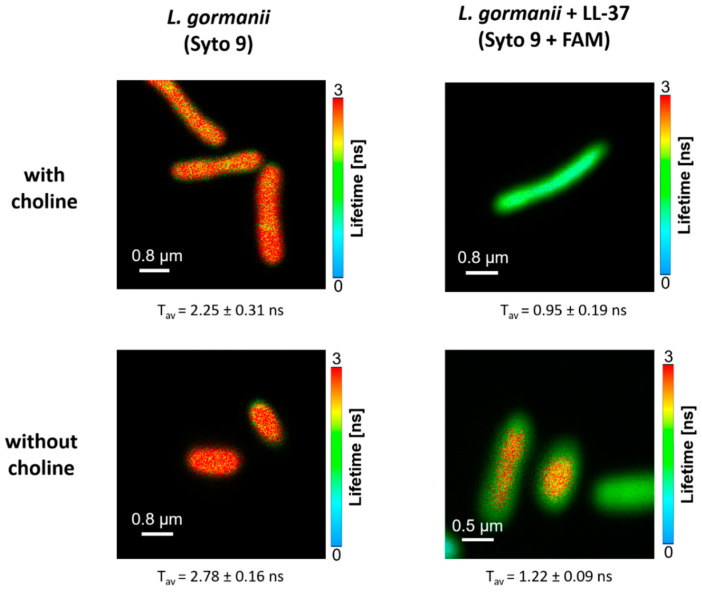
Original FLIM images of the bacteria labeled with Syto 9. The bacteria were either not exposed (**left hand panels**) or exposed (**right hand panels**) to the LL-37 peptide labeled with FAM and grown with (**upper panels**) or without (**lower panels**) exogenous choline. The average fluorescence lifetime values ± S.D. determined from six images for each kind of sample are also reported below the images.

Among the significant spectroscopic and dynamic effect diagnostics for an efficient energy transfer is shortening of the fluorescence lifetime of the donor in the presence of the acceptor. Such a situation was observed in the case of Syto 9 and FAM fluorescence labels situated on the bacterial membranes and the LL-37 protein, respectively (Figure 2B). The fluorescence lifetime situated in the membranes of bacteria, determined to be 2.25 ± 0.31 ns, was shortened to 0.95 ± 0.19 ns after the bacteria were exposed to the FAM-labeled LL-37 peptide. This effect, interpreted in terms of the strong interactions between the LL-37 peptide and the bacteria, can be visible directly from the fluorescence lifetime imaging microscopy (FLIM) images (Figure 3). Interestingly, the magnitude of such an effect depends on whether the bacteria were grown in the presence of exogenous choline (reported above) or without exogenous choline: the change from 2.78 ± 0.16 ns to 1.22 ± 0.09 ns (Figure 3). This difference can be attributed to the influence of bacterial-synthesized phospholipids and their physicochemical properties on the efficient binding of the LL-37 protein to the *L. gormanii* cells.

### 2.3. Surface Pressure—Mean Molecular Area (π−A) Isotherms and Compression Modulus ($C_{S}^{-1}$) Changes

**Figure 4:**
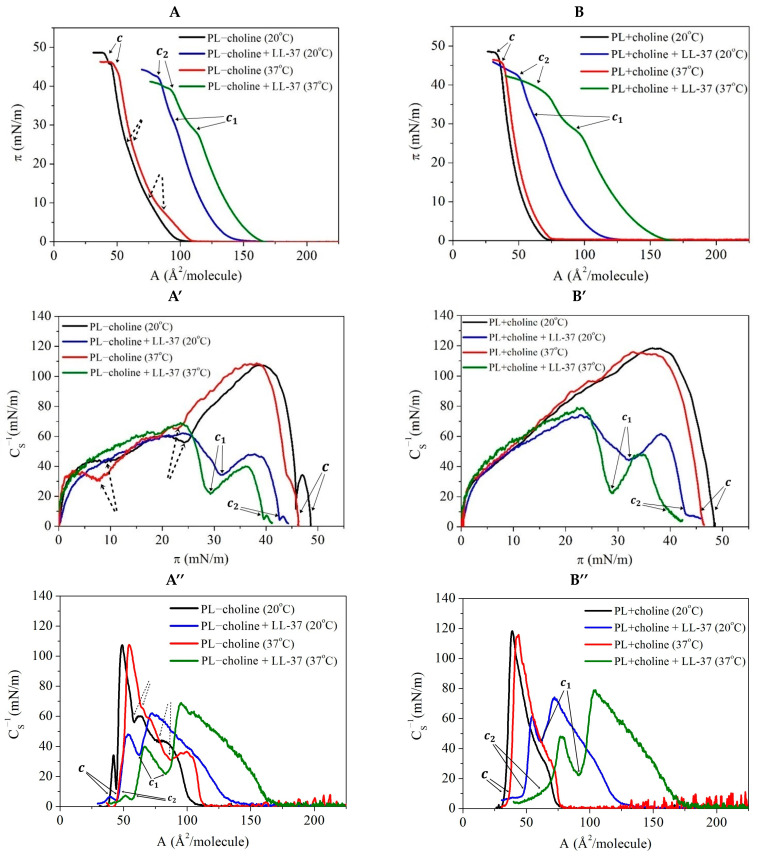
The (**A**,**B**) π−A isotherms and (**A’**,**B’**) compression modulus ($C_{S}^{-1}$) as a function of surface pressure (π) or (**A’’**,**B’’**) mean molecular area (A) obtained for the monolayers composed of phospholipids isolated from the *L. gormanii* bacteria cultured on the (**A**,**A’**,**A’’**) non-supplemented (PL−choline) or (**B**,**B’**,**B’’**) choline-supplemented (PL+choline) medium, in the presence or absence of the LL-37 peptide at 20 °C or 37 °C. The dashed arrows (**A**,**A’**,**A’’**) indicate phase transition locations.

In order to determine the mechanism of the bactericidal action of the LL-37 peptide on the *L. gormanii* model membranes at the molecular level, the Langmuir monolayer technique was used. The Langmuir monolayers composed of phospholipids isolated from the bacteria cultured on the medium without and with exogenous choline were treated with this peptide. During the compression of the PL−choline and PL+choline monolayers, changes in the surface pressure as a function of the mean molecular area (π−A isotherms) resulting from the formation of more condensed and ordered structures were registered. The π−A isotherms obtained for the phospholipid monolayers in the absence or presence of the LL-37 peptide in the subphase at 20 °C or 37 °C are shown in Figure 4A,B. In contrast to PL, the π−A compression isotherm for the acetic acid subphase with LL-37 was not acquired due to the high solubility of the peptide in the 0.01% acetic acid solution. When the PL molecules were present on the subphase, the injected peptide gathered at the interface and interacted with them, affecting the course of the PL compression isotherms. They made it possible to determine the behavior of the PL molecules in the model bacterial membranes affected by the antimicrobial agent.

Based on the π−A compression isotherms, the lift-off area ($A_{0}$) and the collapse pressure ($\pi_{c}$), along with the corresponding area ($A_{c}$), were determined and are listed in Table 1. $A_{0}$ is ascribed to the gas–liquid expanded state transition of the monolayer occurring at π~0.5 mN/m. The collapse pressure ($\pi_{c}$) is the pressure at which the rapid change in the slope of the isotherm takes place. A single collapse point ($\pi_{c}$) is typical of a monophasic or multiphasic mixed system in which all molecules remain at the interface below $\pi_{c}$. Meanwhile, two collapse points can be related to the expulsion of materials to the subphase at lower π (the first collapse pressure, $\pi_{c1}$) or to fracturing, followed by a loss of materials in the subphase or the formation of multilayered aggregates at the liquid–air interface at higher π (the second collapse pressure, $\pi_{c2}$).

Moreover, on the basis of the π−A isotherms, the compression modulus ($C_{S}^{-1}$) known as elasticity was determined for all analyzed monolayers according to the following Equation (1) [13]:

(1) $$C_{S}^{-1}=-A{\left(\frac{d\pi }{dA}\right)}_{T,p}$$

where T means the temperature, and p is the atmospheric pressure.

The compression modulus provides information about the physical state of the monolayer and makes it possible to determine the degree of packing and ordering of molecules on the subphase surface. The $C_{S}^{-1}-\pi$ functions specified for the analyzed systems are presented in Figure 4A’,B’. According to the Davies and Rideal criterion, the $C_{S}^{-1}$ parameter values in the range of 12.5–50 mN/m correspond to the liquid-expanded (LE) state, 100–250 mN/m indicates the liquid-condensed (LC) phase, and the in-between values (50–100 mN/m) point to the intermediate LE–LC state [13,14]. In general, the greater the $C_{S}^{-1}$ value, the more the ordered monolayers with the molecules arranged in a more upright position to the surface plane, resulting in less elastic monolayer formation. To be able to specify the physical state of the *L. gormanii* model membranes, the maximal values of the compression modulus, along with the surface pressure at which they occur, were determined (Table 2). In addition, on the $C_{S}^{-1}-\pi$ plot, the minimum value more accurately identified the surface pressure at which the monolayer collapsed.

The changes in the phospholipid composition caused by the choline presence in the medium for bacteria growth and their influence on the behavior of molecules on the subphase were thoroughly discussed in our previous paper [8]. In summary, the smaller lift-off areas of the π−A isotherms (Figure 4A,B, Table 1) for the PL+choline monolayers suggest that molecules are in closer proximity to each other in comparison to PL−choline. On the basis of the maximal $C_{S}^{-1}$ values (Figure 4A’,B’, Table 2), it was possible to define that, without the peptide, both the PL−choline and PL+choline monolayers are in the liquid-condensed (LC) state, as the $C_{S,max}^{-1}$ at 20 °C and 37 °C exceeds 100 mN/m. For the monolayers without the peptide addition, only one collapse (at $\pi_{c}$) is observed on the π−A isotherms (Figure 4A,B). However, in the case of PL−choline, subtle inflections are noted around 8 mN/m (at 37 °C) or 10 mN/m (at 20 °C) and 25 mN/m (both of) which correspond to two small minima in the course of the $C_{S}^{-1}=f(\pi )$ function (pointed out by the dashed arrows in Figure 4A,A’,A’’). The first inflection can be described as the atypical first-order LE–LC phase transition (domains are visible on the BAM images, Figure S1), as the compression modulus does not decrease to 0 mN/m and the horizontal *plateau* on the π−A isotherms is not noticeable (Figure 4A). The origin of the second inflection is difficult to interpret [8]. However, the further increase in the compression modulus to values greater than for the first local maximum can indicate that molecules are not expelled from the monolayer but that the second-order phase transition related to the changes in alkyl chain tilting takes place, and the monolayer becomes more condensed. The LC phase is revealed by the second local maximum at π~40 mN/m with $C_{S}^{-1}=$ 107 mN/m.

As can be seen in Figure 4A,B and Table 1, the LL-37 peptide addition causes a shift of the π−A isotherms to the right, towards the larger area per molecule at a given temperature, as well as a decrease in the collapse pressure value. Moreover, the temperature increase enhances the LL-37 effect on the phospholipid films. At a lower temperature, the LL-37 addition induces the $A_{0}$ increase by ~47 ${Å}^{2}/molecule$ for the PL−choline and ~51 ${Å}^{2}/molecule$ for PL+choline monolayers. At 37 °C the area changes are equal to ~55 ${Å}^{2}/molecule$ and ~84 ${Å}^{2}/molecule$, respectively (Table 1). This indicates that the PL+choline monolayers are susceptible to the antimicrobial peptide action to a larger extent than the PL−choline model membranes at a higher temperature, while at 20 °C the effect is comparable. Furthermore, the LL-37 peptide addition to the subphase also results in a decrease in the compression modulus maximal values in all the analyzed systems, indicating that the antimicrobial agent causes the phospholipid molecules to form less condensed (more elastic) monolayers at the liquid–air interface (Figure 4A’,B’). The highest $C_{S}^{-1}$ values (Table 2) indicate the intermediate LE–LC phase. The compression modulus dependencies obtained for both PL films with the addition of LL-37 (Figure 4A’,B’,A’’,B’’) are characterized by two maximal values separated by a minimum, which is in agreement with the inflection on the π−A isotherms (Figure 4A,B) and corresponds to the first collapse ($\pi_{c1})$ of the monolayer. At the first maximum, the $C_{S}^{-1}$ values are within 60–80 mN/m (depending on the monolayer type and temperature), while at the second maximum, the values are 14–29 mN/m smaller for PL-choline and 12–31 mN/m for PL+choline, respectively.

Thus LL-37, as the compound with amphipathic properties [15], accumulates at the liquid–air interface and disturbs the LC domain formation due to hindering the reorientation of PL molecules from a horizontal to a vertical position, which leads to elimination of the first-order LE–LC phase transition observed for PL−choline (no distinct domains on the BAM images, Figure S1). One inflection appears on the π−A isotherms at the surface pressure of about 30 mN/m (Figure 4A,B) and correlates with the minimum point on the $C_{S}^{-1}-\pi$ (Figure 4A,B) and $C_{S}^{-1}-A$ curves (Figure 4A’’,B’’). The above value is close to the collapse pressure of the LL-37 monolayer described in the literature, which is in the range of 24–30 mN/m [16,17]. It is commonly known that when two components are immiscible, two collapse states occur which can be ascribed to the collapse surface pressures for the individual pure components [14,18,19]. When the components are partially miscible, some domains enriched mainly by either one or the other component can be formed. Once the surface pressure of collapse of the given component is reached, they collapse and are expelled from the monolayer [14,19]. In this study, the LL-37 or LL-37-enriched phase collapsing at a lower surface pressure should be removed from such PL/LL-37 mixed monolayer at the collapse pressure, which is indicated by Neville et al. as 24–30 mN/m [16,17]. The loss of molecules from the monolayer leads to the smaller degree of its packing revealed by smaller values of the second maximum on the $C_{S}^{-1}-\pi$ curve (Figure 4A’,B’, Table 2). This also correlates with a large reduction in the area per molecule after the first collapse indicating the material desorption from the interface. In contrast, for the monolayers without the peptide, the reduction in the mean molecular area during the compression is accompanied by an increase in the packing density of the monolayer as evidenced by the increase in the compression modulus values (Figure 4A’’,B’’).

Furthermore, in the presence of the peptide, the second collapse pressure ($\pi_{c2})$ values are smaller, which proves that the monolayers are less stable than the PL monolayers without the LL-37 in the subphase. The above statements and observations confirm that due to the complex interactions, the cathelicidin changes the PL molecules’ behavior on the subphase surface and evokes the destabilization of both monolayers.

### 2.4. Surface Potential Changes—Mean Molecular Area (∆V−A) Isotherms

**Figure 5:**
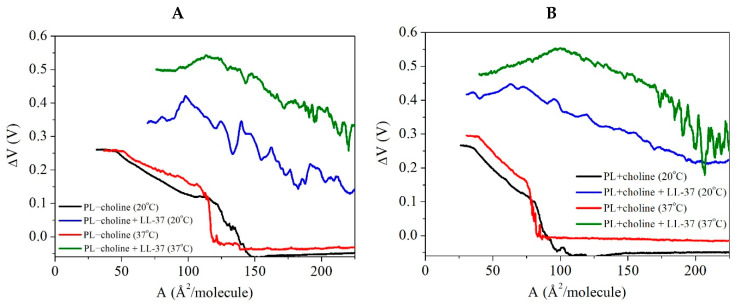
The surface potential changes (∆V) as a function of mean molecular area (A) obtained for the monolayers composed of phospholipids isolated from *L. gormanii* bacteria cultured on the (**A**) non-supplemented (PL−choline) or (**B**) choline-supplemented (PL+choline) medium, in the presence or absence of the LL-37 peptide at 20 °C or 37 °C.

Along with the surface pressure, the surface potential changes (∆V) were measured. Phospholipids, as amphiphilic compounds, contain two (or four in the cardiolipin molecule) hydrocarbon chains, orienting away from the polar subphase and hydrophilic head group, which interact strongly with the liquid phase [20]. When the phospholipids are far away from each other (at large values of the mean molecular area), the ∆V is fairly constant. Upon compression of the PL molecules at the liquid–air interface, their orientation changes, due to the increasingly stronger intermolecular interactions resulting from the molecular distance decrease. Formation of the more ordered and condensed structures evokes a change in the normal component of the dipole density, which is observed as the ∆V increase [20]. The maximal value corresponds to the most packed state of the monolayer in which molecules assume an orientation as vertical as possible [21]. Therefore, the measured values of the surface potential changes make it possible to analyze the orientation of molecules on the subphase surface [21]. The surface potential changes of the *L. gormanii* phospholipid monolayers with or without the peptide are presented as the ∆V−A isotherms in Figure 5.

As follows from Figure 5, the maximal ∆V values are comparable and reach around 0.27–0.30 V for the pure PL−choline and PL+choline monolayers, both at 20 °C and 37 °C. The addition of the LL-37 peptide affects both films to a large extent, as the significant surface potential increase is observed for the PL+choline and PL−choline monolayers. The course of the ∆V−A dependencies is similar, and the maximal values are approximately the same at a given temperature. In the early stages of compression, the ∆V−A curve is characterized by many fluctuations that are more pronounced in comparison to the films without the peptide.

As the phospholipid molecules are applied to the surface with LL-37 already on it and the intermolecular interactions with PLs can already take place, the ∆V values are bigger from the early stages of compression. It is worth mentioning that the model membranes in the presence of the peptide at 20 °C and 37 °C obtain the greatest ∆V values near the monolayer’s first collapse (Figure 4). Above that point, as the peptide-rich phase is pushed out of the monolayer, the molecules are less densely packed on the surface, which leads to a greater inclination of the molecules with respect to the subphase plane and, consequently, to a decrease in the surface potential (Figure 5).

### 2.5. Surface Morphology

**Figure 6:**
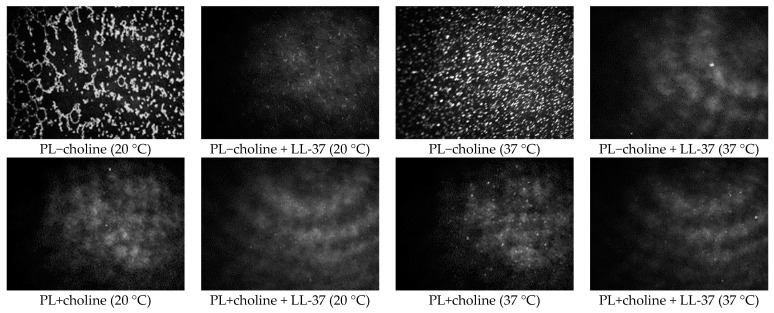
BAM images (720 μm × 400 μm) taken at 30 mN/m for the monolayers composed of phospholipids isolated from the *L. gormanii* bacteria cultured on the non-supplemented (PL−choline) or choline-supplemented (PL+choline) medium, in the presence or absence of the LL-37 peptide at 20 °C or 37 °C.

The Brewster angle microscopy technique was used to observe the morphology of the model membrane surface. As aforementioned, the composition of the phospholipid mixtures and the specific interactions between the components can lead to formation of at least two-phase structures, referred to as domains [22]. They occur naturally in the bacterial membranes in order to minimize the system energy; however, the presence of antibiotics or other antimicrobial substances could also alter or induce the membrane components’ sequestering [23]. The images of PL−choline and PL+choline surface morphology under the various conditions are presented in Figure 6. The images were taken at π~30 mN/m, as this value of surface pressure corresponds to the equilibrium lateral pressure of biomembranes [24]. The data obtained at the other values of surface pressure are included as the Supplementary Data (Figures S1 and S2).

The morphology of the PL model membranes without the peptide addition was analyzed thoroughly in our previous paper [8]. The results showed greater homogeneity of PL+choline, as it comprises mostly zwitterionic phospholipids, in comparison to the PL−choline monolayers containing a larger amount of negatively charged molecules, promoting phase separation.

In all the analyzed monolayers, the LL-37 peptide addition results in more homogeneous monolayers. The greatest difference is visible for the PL−choline films, due to the high heterogeneity and large areas of condensed structures observed in the absence of the antimicrobial agent. The domains formed in the presence of LL-37 are small, circular, and occur in relatively minor quantities, even at great values of surface pressure.

Taking into account the LL-37’s positive charge, it is justified to assume that the peptide interacts preferentially with the anionic PLs from the early stages of compression, thus modifying the surface morphology and limiting domain creation by decreasing the repulsive interactions among the PL molecules. The reduction in the phospholipid domain amount caused by the LL-37 peptide was also reported by Majewska et al. [25]. Moreover, at a higher temperature, the domains are less numerous compared to 20 °C, possibly as a result of the thermal motion of molecules. Disruption of the natural domain structure of the bacterial membranes can be responsible for the peptide antimicrobial effect.

### 2.6. Penetration Study

Monolayer penetration studies are widely used for investigating the interactions between model membranes and peptides, antibiotics, or other biomolecules. The experiments can be performed at constant area value or constant surface pressure value [26]. The peptide penetration of the phospholipid monolayers in the presented studies was investigated at the constant surface pressure of 30 mN/m, to better mimic the LL-37 influence on the bacterial membranes in human organism. This value is reported to be consistent with the molecular density occurring in the bilayers [27] and the equilibrium lateral pressure established for the biomembranes [24]. As the surface pressure is kept constant, the mean molecular area (A) changes describe the monolayer−peptide interactions. The increase in the area value indicates incorporation of the peptide into the PL film, resulting in an expansion of the monolayer. On the contrary, the A decrease follows from the desorption of molecules from the monolayer into the subphase [28]. This allows one to specify the effect of, e.g., antimicrobial agent on bacterial membranes.

It is important to point out that for obtaining the π−A isotherms, the peptide was dropped on the subphase surface before the phospholipids were applied; thus, the LL-37 could interact with the unorganized PL molecules widespread throughout the surface. To the contrary, in the penetration studies, the peptide is injected under the compressed monolayer; therefore, the effect of LL-37 on the intermolecular interactions in the PL films can be significantly different. Nevertheless, both methods complement each other, allowing for the assessment of differences in the behavior of PL molecules in the monolayers due to the peptide presence. While all of the performed analyses provide a lot of information on the molecular behavior and lead to more detailed characteristics of the model membranes, the penetration studies seem to reflect better the bacterial cell response to the LL-37 peptide presence in the human organism. Therefore, the registered data provide adequate information considering the *L. gormanii* response to the peptide occurrence.

**Figure 7:**
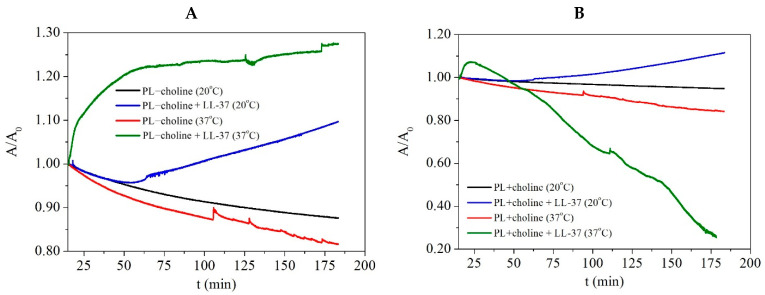
The normalized mean molecular area changes ($A/A_{0}$) in time (t) obtained for the monolayers composed of phospholipids isolated from the *L. gormanii* bacteria cultured on (**A**) the non-supplemented (PL−choline) or (**B**) the choline-supplemented (PL+choline) medium, in the presence or absence of the LL-37 peptide at 20 °C or 37 °C.

After compressing the monolayer to the surface pressure of 30 mN/m, the alterations of the mean molecular area were monitored for about 3 h. The obtained results were normalized ($A/A_{0}$) and expressed as a function of time in Figure 7. In the measurements with LL-37, the peptide was added after about 15 min of the monolayer equilibration at the given surface pressure. Thus, only the data registered after this time are considered in the further analysis.

**Figure 8:**
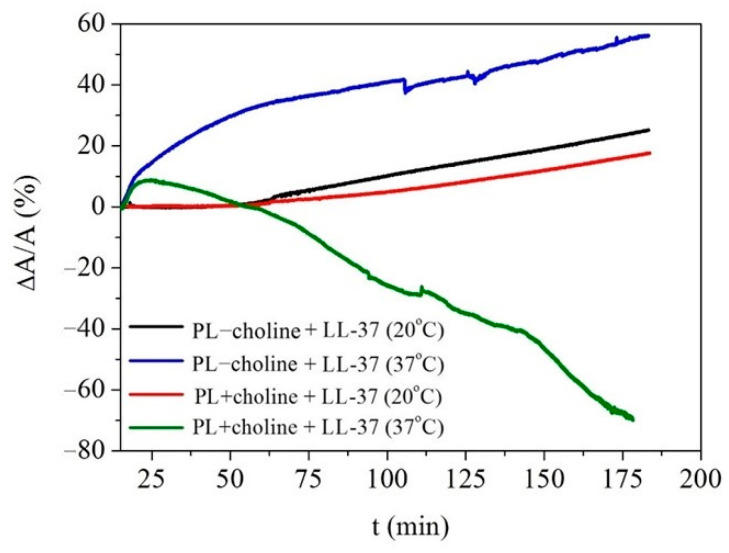
The relative area change (∆A/A) as a function of time (t) for the PL−choline and PL+choline monolayers at π = 30 mN/m caused by the addition of LL-37 peptide into the subphase at 20 °C or 37 °C.

In general, the $A/A_{0}$ decrease can be noted in the pure PL monolayers, indicating instability of the obtained films at the given surface pressure due to the desorption of molecules. After the 3 h equilibration, the area reduction is 12% and 18% for PL−choline and 5% and 16% for PL+choline at 20 °C or 37 °C, respectively. On the other hand, the increase in the mean molecular area is caused by the penetration of the peptide into the monolayer. In order to determine the area changes induced by the addition of the peptide, the relative area change (∆A/A) was calculated as the difference between the mean molecular area recorded in the presence and absence of LL-37 at the given surface pressure value, in relation to that occupied by the molecule in the pure PL monolayers. These changes are expressed in percentages (Figure 8).

For comparative purposes, the ∆A/A values obtained for the films after ~3 h from the peptide injection were taken into consideration. They are presented in Table 3. As already mentioned, the greater mean molecular area (∆A/A > 0) due to the barrier expansion at the constant surface pressure indicates the peptide’s incorporation into the phospholipid monolayer [17], while the opposite phenomena (∆A/A < 0) can point out the molecules’ expulsion into the subphase or molecular reorganization occurring on the subphase surface [16].

At 20 °C, the effect of the LL-37 peptide on the PL−choline and PL+choline monolayers is similar, and no significant differences in the PL molecules’ behavior are observed between the two. This is revealed in the comparable π−A isotherm shifts (Figure 4A,B, Table 1) caused by the antimicrobial agent presence as well as the similar compression modulus decrease (Figure 4A’,B’,A’’,B’’, Table 2). In the penetration study, after the peptide addition, no effect is noted up until 50 min—the area decrease is identical to that of the pure phospholipid films (Figure 7). Subsequently, an increase is observed, and after 3 h from the injection, the molecular area still rises, and no *plateau* occurs. Therefore, it can be assumed that the LL-37 peptide incorporates gradually into the film and alters the PL molecules’ organization on the subphase surface. No abrupt changes in the mean molecular area occur, and the effect of LL-37 (Figure 8) is only slightly greater for PL−choline (∆A/A = 25%) than for PL+choline (∆A/A = 18%).

It is of significant importance to note that measurements conducted at 37 °C better reflect the processes taking place in the human organism, as it is the physiological temperature of the body. For PL+choline, at first a 9% area increase is observed (Figure 8), which makes it possible to conclude that the peptide incorporates into the PL monolayer immediately after the injection. A similar conclusion was drawn by Sevcsik et al. [17]. Then, a significant area decrease is observed as the ∆A/A reaches −70%, suggesting that the so-called critical destabilization [16] occurs just 10 min after the LL-37 addition. On the contrary, the PL−choline analysis at this temperature showed that the area increases immediately after antimicrobial agent insertion, keeps rising, and then reaches a *plateau*. Some fluctuations can be still noted; however, it can be due to the high kinetic energy of the molecules as well as the subphase evaporation. The ∆A/A is equal to 56%; thus, the peptide is built into the phospholipid film. However, it does not result in great destabilization, as was observed in the case of PL+choline. The penetration results indicate that the PL+choline films are more susceptible to the LL-37 action in comparison to PL−choline at a higher temperature, which is consistent with the π−A isotherm courses (Figure 4A,B) and the compression modulus decrease (Figure 4A’,B’,A’’,B’’).

## 3. Discussion

The successful colonization of lung tissue by the *L. gormanii* bacteria can be attributed to their ability to produce enzymes that degrade a lung surfactant, an essential component in maintaining proper lung function and protecting against incoming pathogens. One of the major components of the lung surfactant is PC, which can serve as a substrate for the phospholipase PlaB produced by *L. gormanii* [29]. Utilizing extracellular choline to synthesize PC, *L. gormanii* modifies the quantitative composition of membrane lipids, resulting in the alteration of the physicochemical properties of the membrane and its interactions with antibacterial peptides. Our previous studies demonstrated that LL-37 possesses a bactericidal action against *L. micdadei*. The bacteria cultured on the medium supplemented with choline displayed increased susceptibility to this peptide compared to the bacteria cultured without the addition of exogenous choline. The peptide perturbed bacterial-derived phospholipid monolayers, and the interactions were dependent on the molar portion of PC [30]. LL-37 binds to the membrane of *L. gormanii* as demonstrated by fluorescence lifetime microscopy, and the interaction was stronger with the bacteria cultured on the medium supplemented with exogenous choline. Compared to *L. micdadei*, LL-37 exhibits a more potent bactericidal effect on *L. gormanii* as a result of its higher efficacy at a lower concentration. However, the concentration of 10 µM LL-37 resulted in an approximately 50% killing rate of *L. gormanii* in the live/dead assay, whereas in the plate assay, it showed a higher killing rate of 83% and 91% for the bacteria cultured on the medium without and with exogenous choline, respectively, surpassing the effect observed with the 20 µM concentration of LL-37. The probable cause of these differences is the transition of the *L. gormanii* bacteria into a viable but non-culturable state (VBNC) under the influence of 10 µM LL-37. The confirmation of this assumption is supported by the fact that after plating the LL-37-treated bacteria at the concentration of 10 µM onto the BCYE medium with sodium pyruvate, the number of grown colonies was larger compared to that of the colonies cultured on the standard medium. Despite sodium pyruvate (SP) being a well-known intermediate key metabolite in glycolysis and proven to facilitate the resuscitation of VBNC cells [31], the exact mechanism underlying the transition of *L. gormanii* bacteria into a cultivable state on the artificial medium remains to be elucidated.

To investigate the mechanism of the bactericidal action of the LL-37 peptide on *L. gormanii*, Langmuir monolayers composed of phospholipids isolated from the bacteria cultured on the medium without and with exogenous choline were exposed to this peptide. The analysis of changes in the membrane’s physiochemical properties made it possible to propose the mechanism of antimicrobial agent action. Our previous study showed a substantial difference in the phospholipid composition of the *L. gormanii* membranes resulting from the presence of exogenous choline in the bacterial medium [7]. These various proportions of phospholipids influence not only the physicochemical properties of the model membranes composed of the four PL classes [8], but also the interactions with the human cathelicidin LL-37.

Considering all the results obtained for the PL−choline and PL+choline monolayers at 20 °C and 37 °C with or without the peptide addition, several conclusions can be drawn. The pure PL+choline films are more tightly packed and ordered than PL−choline, which is highlighted by the values of the compression modulus (Figure 4A’,B’,A’’,B’’, Table 2). The PL−choline films are characterized by a larger content of anionic PLs (24%), thus stronger repulsive forces occur between the monolayer components resulting in the more expanded monolayer [8]. At a higher temperature, due to the great kinetic energy of the molecules and thermal motions, these interactions become even stronger. On the contrary, PL+choline, comprising only 15% of negatively charged and 85% of zwitterionic lipids, are characterized by more attractive forces (reduced by a higher temperature), therefore the formed monolayers are more rigid. These differences are also reflected in the surface morphology (Figures S1 and S2). In all analyzed monolayers, the peptide presence leads to a decrease in the compression modulus (Figure 4A’,B’,A’’,B’’) as well as to a shift of the π−A isotherms (Figure 4A,B) towards the greater values of the mean molecular area. These dependencies are more pronounced for the PL+choline films, indicating their greater susceptibility to the LL-37 action.

**Figure 9:**
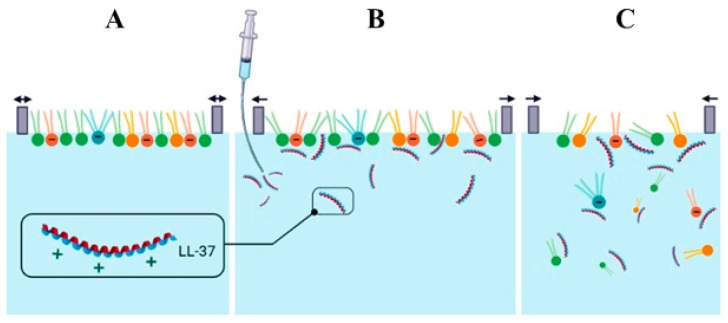
The possible mechanisms of LL-37 action on the *L. gormanii* model membranes: (**A**) equilibration of the PL monolayer—balance (↔); (**B**) changes in orientation of the PL molecules and incorporation of the peptide into the monolayer—expansion (←→); (**C**) membrane disruption and expulsion of the PL molecules into the subphase—compression (→←). The hydrophobic peptide residues are schematically designated in red.

To develop a mechanism for the LL-37 peptide action on the model membranes, numerous factors need to be taken into account. Many authors describe the great affinity of cationic LL-37 for the anionic phospholipids in the monolayers and bilayers, which is believed to be crucial for the peptide activity towards the bacterial membranes [16,17,26,32]. In principle, a larger content of anionic lipids in the PL−choline model membranes would suggest that these films will be more susceptible to the LL-37 peptide. Meanwhile, our studies proved the opposite effect at 37 °C, where the PL+choline monolayers exhibit the largest destabilization in the presence of the peptide. Therefore, other possibilities should be considered (Figure 9).

The cationic peptide molecules are drawn towards the anionic phospholipids and interact strongly with the hydrophilic head groups. Furthermore, the charge of the monolayer defines the LL-37 organization in the subphase, as it can remain in the oligomeric or monomeric form [24,33,34]. These phenomena lead to the alterations of the PL molecules’ orientation and overall monolayer ordering. At 20 °C, a slightly larger PL−choline area increases compared to the PL+choline (Figure 8), which most likely results from the higher percentage of negatively charged lipids present in the PL−choline film, as the affinity of the positively charged peptide for PG and CL promotes incorporation. The LL-37 penetration into the PL−choline monolayer is more pronounced at a higher temperature, in comparison to 20 °C, as the molecules have greater kinetic energy and are more mobile. Therefore, the monolayers are slightly disordered due to the constant movement of the molecules, and the LL-37 peptide can incorporate into the monolayer more readily and rapidly [35], which is observed as a sudden mean molecular area increase after the peptide injection (Figure 7).

As already mentioned, in the presented studies, the greatest monolayer destabilization is observed for PL+choline analyzed at 37 °C and, contrary to the less condensed PL−choline monolayer exposed to the LL-37 peptide, the drastic area decrease takes place (Figure 7 and Figure 8). Interestingly, PL+choline contains large amounts of PC [7], which in many studies did not show significant interactions with LL-37 [16,32]. These results indicate that not only electrostatic but Lifshitz–van der Waals interactions can define the peptide mechanism of action as well. Moreover, the presence of hydrogen bonds cannot be ruled out either [36]. Naturally, both types of interactions (polar and non-polar) can occur in the analyzed systems; nevertheless, their share and strength determine the monolayer’s behavior under the LL-37 influence. The favorable electrostatic interactions drive the initial peptide anchoring [36]. Large affinity and strong attraction for the head groups keep the LL-37 peptide in the head group region, preventing the peptide from penetrating more deeply towards the hydrophobic chains. Hydrogen binding with the PL molecules allows aggregation on the membrane surface [36]. As a consequence, no critical destabilization occurs, and the peptide’s incorporation into the PL−choline monolayer is observed (Figure 9B). If the cationic compound, in this case LL-37, is hydrophobic enough, it can penetrate more deeply into the monolayer and interact with the fatty acid chains [37,38], rather than just with the polar head groups. The stronger the electrostatic LL-37−PL head group attraction, the smaller depth the peptide can penetrate [35]. As the PL+choline monolayer does not contain as many anionic compounds, LL-37 passes through the zone of the polar groups. Then the peptide can interact with the fatty acid (FA) chains, causing severe alterations in the molecular orientation and resulting in critical destabilization as well as expulsion of the molecules into the subphase (Figure 9C).

The above mechanism indicates that, in fact, the LL-37 peptide is able to interact and penetrate the monolayers with a large content of zwitterionic PLs to a great extent. This is consistent with the studies by other researchers showing that the antimicrobial agent interactions with the FA chains are the factor responsible for the monolayers’ disruption [35]. Therefore, the mechanism of the antimicrobial peptide action is dependent on the composition of the model membrane. Thus, it can be stated that the PC-enriched PL+choline monolayers are more susceptible to the LL-37 peptide, compared to the more anionic PL−choline, due to the larger share of Lifshitz–van der Waals interactions decisive for membrane disruption.

The effect of LL-37 on the *L. gormanii* model membranes is much stronger than on those of *L. micdadei* [30] and *L. dumoffii* [39] at the same peptide concentration in the bulk phase (0.08 μg/mL). This is revealed in the monolayer expansion confirmed by the shifts of the π−A isotherms towards larger mean molecular areas. For instance, the changes in the lift-off areas determined for the PL−choline and PL+choline monolayers in the presence of LL-37 are 21% and 23% for *L. micdadei* [30], 18% and 28% for *L. dumoffii* [39], and 48% and 72% for *L. gormanii*. Thus, the observed changes in the area for the *L. gormanii* model membranes are two to three times greater than in the other membranes, indicating the strongest disruptive effect. This is also manifested in a significant decrease in the degree of packing and ordering of the membranes as indicated by the maximal compression modulus values. The percentage decreases are as follows: 0% and 21% for *L. micdadei* [30], 27% and 32% for *L. dumoffii* [39], and 42% and 38% for *L. gormanii*, in the absence or presence of exogenous choline, respectively. Additionally, the effect of the peptide on the PL+choline membranes was found to be greater than on PL−choline. This is due to the compositional differentiation in these three *Legionella* species caused by the choline addition to the growth medium [30,39]. It can be concluded that the negative charge of PL molecules does not play an exclusive role in the susceptibility of *Legionella* bacteria membranes to the positively charged peptide, but such a role can also be assigned to the Lifshitz–van der Waals interactions between the LL-37 and FA chains, which leads to a strong fluidization of the membranes. These studies demonstrated that the LL-37 peptide exhibited a bactericidal effect on *L. gormanii* cells by binding to their membrane and subsequently penetrating it. Moreover, the interaction between the LL-37 peptide and the *L. gormanii* membrane was found to be more pronounced at 37 °C compared to 20 °C.

The exploration of novel bactericidal compounds holds significant importance, especially considering the prevalence of antibiotic resistance genes like aminoglycoside phosphotransferase (APH(9)-Ia), Ambler class B metallo-β-lactamase (FEZ-1), and tetracycline-resistant ribosomal protection protein (tet56) in the genome of *L. gormanii* [4]. The phenotypic plasticity of bacterial membranes plays a crucial role in determining susceptibility to antimicrobial peptides, which are an integral part of host defenses. This feature holds significant implications for the design and evaluation of innovative therapeutic strategies aimed at treating and preventing bacterial infections. Antimicrobial peptides, initially derived from natural sources, serve as the foundation for developing synthetic analogs with enhanced efficacy, prolonged duration, and minimal adverse effects to address the potential issues encountered with drug candidates. Designing the sequence of these molecules and utilizing advanced bioinformatics methods and computational simulations, based on natural peptides like the LL-37 peptide, will enable effective combat against human pathogens such as *Legionella*.

## 4. Materials and Methods

### 4.1. Live/Dead Bacteria Staining Assay

*L. gormanii* (ATCC 33297) was cultured on the buffered charcoal yeast extract (BCYE) agar (Oxoid, Basingstoke, UK) and on this medium supplemented with 100 μg/mL choline chloride (Sigma-Aldrich, St. Louis, MO, USA) at 37 °C and 5% CO_2_ in the humid atmosphere for 3 days. Next, 50 µL of the LL-37 peptide solution [LL-37 (human) trifluoroacetate salt, Merck, Darmstadt, Germany; purity ≥ 95%] at the final concentrations of 10 µM and 20 µM was added to 50 µL of the bacterial suspension in water with OD_600_ = 0.1, and incubated for 1 h at 37 °C. The suspension was transferred into a sterile Eppendorf tube and stained using the Live/Dead BacLight bacterial viability assay kit (ThermoFisher, Waltham, MA, USA). The bacterial suspension was stained in shielded tubes to minimize light exposure using 5 µM Syto9 and 30 µM propidium iodide in 0.3% DMSO for 15 min at room temperature. The live/dead stained images of the LL-37 peptide-treated and untreated bacteria (control) were collected using the Axiovert 200 M confocal microscope with the LSM 5 PASCAL scanning head (LSCM) (Carl Zeiss, Jena, Germany). The cells were counted from 20 microscopic images of each test sample. The images were captured with the AxioVision 4.8 software (Carl Zeiss) in the multichannel fluorescence technique, with the AxioCam HR3 camera (Carl Zeiss, Jena, Germany), using 470 nm and 546 nm filters for the green and red channels, respectively, with the same exposure time for each pair of images. The quantification of live/dead was performed with ImageJ 1.50i (Wayne Rasband, National Institutes of Health, Kensington, MD, USA). The sum of the lighting values was analyzed separately for each channel in the pair of images, which corresponds to the percentage of live and dead bacteria.

### 4.2. Colony Forming Unit (CFU)—Counting Assay

*L. gormanii* was cultured on BCYE agar (Oxoid, Basingstoke, UK) with and without 100 μg/mL choline chloride (Sigma-Aldrich, St. Louis, MO, USA) at 37 °C and 5% CO_2_ in the humid atmosphere for 3 days. The bacteria were suspended in sterile MilliQ water (OD_600_ = 0.1), and the subsequent 10-fold dilutions were performed to 10^−3^. Then, 5 µL of the last dilution of the bacterial suspension was transferred into a sterile Eppendorf tube, mixed with 5 µL of sterile MilliQ water (control) or the LL-37 peptide solution in water (10 µM and 20 µM). After 1 h of incubation at 37 °C, the bacterial suspensions were plated on the BCYE medium and incubated for 3 days at the same temperature. Subsequently, the number of CFUs was enumerated from the BCYE plates. The studies were carried out in three independent experiments, with each experiment including three replicates.

### 4.3. Fluorescence Lifetime Imaging Microscopy

The FRET (Förster resonance energy transfer) spectroscopy technique was employed to investigate the interactions between *L. gormanii* and the LL-37 peptide. The study utilized the MicroTime 200 microscopic confocal fluorescence system provided by PicoQuant GmbH (Berlin, Germany). In this experiment, the LL-37 peptide was labeled with FAM (carboxyfluorescein, ANASPEC, Seraing, Belgium, purity ≥ 95%), while the bacteria were labeled with Syto9 dye (Thermo Fisher Scientific, Waltham, MA, USA). A total of 50 µL of the 5-FAM-LL-37 peptide solution at the final concentration of 20 µM was added to 50 µL of the bacterial suspension in water with OD_600_ = 0.1, while 20 µL of the sample was applied on the microscope coverslips coated with polylysine, which aided in immobilizing the bacteria. To initiate the excitation, energy transfer process, and fluorescence, a pulsed laser beam operating at the wavelength of 440 nm was directed onto the sample through a dichroic mirror (ZT442rdc-UF3 Chroma) and a high numerical aperture objective (Olympus 100×, NA 1.3). The emitted fluorescence was collected by the same objective in the confocal mode using a 50 μm diameter pinhole. Only the donor fluorescence (Syto9) was collected, distinguished from the total emission signal through the combination of a dichroic beam splitter (FF495-Di03-25 × 36 Semrock) and a 465 long-pass filter (AT465lp Chroma) placed in front of the avalanche photodetector (Excelitas Technologies, Waltham, MA, USA). The required mirrors for the set-up were provided by Analysentechnik (Mainz, Germany).

The excitation laser was operated in the pulse mode with a repetition rate of 10 MHz, and the system was set to the single-channel lifetime resolution of 16 ps. Photon detection was accomplished using the time-correlated single photon counting mode facilitated by a TimeHarp 400 board. The acquired data were subsequently analyzed using the SymPhoTime software package (version 2.3) developed by PicoQuant in Germany. The intensity decays (*I*(*t*)) for each sample were deconvoluted using the multi-exponential model expressed as

(2) $$I\left(t\right)=\sum_{i}\alpha_{i}exp\left(-\frac{t}{\tau_{i}}\right)$$

where *τ_i_* represents the decay times, and *α_i_* denotes the pre-exponential factors. Then, the average lifetime ($\langle \tau \rangle$) was calculated using the amplitude-weighted formula:

(3) $$\langle \tau \rangle =\frac{\sum_{i}\alpha_{i}\tau_{i}}{\sum_{i}\alpha_{i}}$$

The Förster distance (R_0_ = 57 Å) was calculated based on the spectral properties of the Syto9 and FAM dyes. The steady-state fluorescence measurements were performed utilizing a Shamrock 163 spectrograph, which was coupled to a MicroTime 200 microscope set-up, enabling direct spectral analysis of individual bacterial specimens. The detection system employed a Newton EMCCD DU970P BUF camera (manufactured by Andor Technology, Belfast, UK) that was cooled to the temperature of −50 °C. A Cary 60 UV-VIS spectrometer, developed by Agilent Technologies, was employed for the absorption spectra acquisition.

### 4.4. Extraction of Lipids

Lipids were extracted from the freeze-dried bacterial mass of *L. gormanii* grown on the medium with and without the addition of exogenous choline using the Bligh and Dyer (1959) method [40]. Briefly, the bacterial mass (450 mg) was suspended in 34 mL chloroform/methanol (1/2; *v*/*v*) and vigorously mixed using a magnetic stirrer for 4 h at room temperature. Next, the suspension was centrifuged for 30 min, 6000× *g*, 4 °C. The organic phase was collected, and a new portion of chloroform/methanol (1/2; *v*/*v*) was added to the bacterial pellet. Lipid extraction was continued for 3 h. After the re-centrifugation with the parameters as laid out above, organic phases were pooled, and chloroform and water were added to the final methanol/chloroform/water ratio of 2/2/1.8 (*v*/*v*/*v*). After thorough mixing of the reagents and centrifugation for 30 min, 6000× *g*, 4 °C, the lower organic phase was collected and dried using a rotary evaporator (Rotavapor R-100). Next, the samples were suspended in a mixture of hexane/isopropanol (3/2; *v*/*v*), mixed thoroughly, and centrifuged for 20 min, 6000× *g*, 4 °C. The lipid-containing organic phase was dried under a nitrogen stream and stored at −80 °C for further analysis. *L. gormanii* cultured without exogenous choline yielded 27 mg lipids (6% dried biomass). The bacteria cultured with the exogenous choline supplementation produced 31.5 mg lipids (7% dried biomass).

### 4.5. Separation of Phospholipids into Classes by Thin-Layer Chromatography

Before the chromatographic separation of phospholipids into classes, one-dimensional thin-layer chromatography (TLC) was performed to purify the extracted lipids from the pigments, mainly legioliulin, which exhibits the blue-white autofluorescence under long-wavelength UV [41]. Chromatographic separations were carried out on the silica gel 60 F254 plates of the size 10 cm × 10 cm (Merck, Darmstadt, Germany). The plates were washed with chloroform/methanol (1/1; *v*/*v*) prior to use in order to remove all contaminants and air-dried at room temperature. Lipids (about 2 mg) resolved in 20 μL of chloroform/methanol (1/2; *v*/*v*) were applied to the silica gel as a narrow band and developed with the solvent system chloroform/methanol/acetic acid (98/2/1; *v*/*v*/*v*). The lipids were visualized with iodine vapor, and pigments were detected under long-wavelength UV (Transiluminator UV-953). The lipid-containing band was scraped off, transferred to the screw-capped tubes, and extracted from the silica gel with a mixture of chloroform/methanol (1/1; *v*/*v*). PLs purified from the pigments were intended for the Langmuir monolayer study.

### 4.6. Langmuir Monolayer Study

The phospholipids isolated from the *L. gormanii* bacteria cultured on the non-supplemented (PL−choline) or choline-supplemented (PL+choline) medium were dissolved in chloroform (Avantor Performance Materials Poland S.A., Gliwice, Poland; purity > 99.9%) and methanol (ROMIL Chemicals Ltd., Cambridge, UK; purity > 99.9%) at the ratio of 4/1 (*v*/*v*). 1 mg/mL solutions were obtained. Langmuir–Blodgett (KSV 2000 Standard, KSV Instruments, Helsinki, Finland) and Langmuir (KSV Nima, Biolin Scientific, Stockholm, Sweden) troughs equipped with symmetric barriers and a Wilhelmy plate for the surface tension determination (with the 0.1 mN/m accuracy) were employed for the surface pressure–molecular area (π−A) isotherm measurements. The 0.01% acetic acid solution used as a subphase was prepared by diluting the concentrated one (Avantor Performance Materials Poland S.A., 99.7%) with water purified by the Milli-Q system (resistivity 18.2 MΩcm). An external water circulating system (Lauda, Schwechat, Austria) was used to keep a constant temperature (20 °C or 37 °C; ± 0.1 °C) during the measurements.

The prepared phospholipid solutions were applied to the subphase surface by means of a Hamilton microsyringe. To ensure that the solvents were fully evaporated, the trough was left for 10 min. Then, the monolayer was compressed with the barrier rate of 10 mm/min until the film collapsed. Each experiment was repeated 2–3 times, giving the mean error of ±2 ${Å}^{2}$/molecule. The π−A isotherms for the PL monolayers with the addition of the LL-37 peptide (LL-37 (human cathelicidin) trifluoroacetate salt, Sigma-Aldrich, St. Louis, MO, USA; purity ≥ 95%) to the subphase were obtained by dropping 50 μL of the peptide solution into 0.01% acetic acid (1 mg/mL) onto the subphase surface, giving a final concentration of 0.08 μg/mL in the bulk phase, leaving it for two hours and then applying the phospholipid solution and carrying on as described above. Acetic acid was employed to keep the peptide’s structure while in the solution. Simultaneously, the surface potential changes were recorded with 1 mV accuracy as a function of area (the ∆V−A isotherms) by means of a surface potential sensor (SPOT, Biolin Scientific, Stockholm, Sweden). Moreover, to directly visualize the monolayer’s morphology, a Brewster angle microscope (nanofilm_ultrabam, Accurion, Göttingen, Germany) was used. A solid-state 50 mW laser, emitting *p*-polarized light (wavelength = 658 nm), was employed, and the incident angle was 53.2°.

To be able to determine the monolayer penetration by the peptide, the experiments were conducted as follows. The phospholipid solution was applied to the subphase, and after the solvent’s evaporation, the obtained monolayer was compressed up to the surface pressure of 30 mN/m. Then, the compression was stopped, the monolayer was equilibrated at the constant surface pressure, and, after 15 min, the peptide solution was injected into the subphase, underneath the monolayer and the barrier level. After that, the changes in the mean molecular area were observed for about 3 h. As a control, the measurement without the peptide addition was performed analogously for the same period of time.

## 5. Conclusions

The human cathelicidin LL-37 exhibited a dose-dependent bactericidal effect on *L. gormanii* cells. At a concentration of 10 µM, the LL-37 peptide rendered the bacteria viable but non-culturable. The antibacterial activity of the LL-37 peptide was attributed to its strong binding to the bacterial membrane, as evidenced by the fluorescence lifetime imaging microscopy. The changes in the phospholipid composition of *L. gormanii* cells, caused by the exogenous choline presence, altered the model bacterial membrane response to the LL-37 peptide significantly. Depending on the zwitterionic and anionic phospholipid contents, two mechanisms of the LL-37 action were proposed. Both the polar (electrostatic and hydrogen bonding) and non-polar (Lifshitz–van der Waals) interactions were found to play a pivotal role in the molecular mechanism of the peptide operation at the interface. PL+choline was more susceptible to the LL-37 antimicrobial action than PL−choline, since, at 37 °C, the intermolecular LL-37−PL interactions led to extensive monolayer disruption. A larger content of negatively charged PLs in PL−choline provoked the peptide binding into the film’s head group region, due to the strong polar interactions. To the contrary, large amounts of zwitterionic compounds (PL+choline) resulted in critical membrane destabilization, as a result of dominant Lifshitz–van der Waals forces between the peptide hydrophobic parts and the acyl chains of phospholipids.

## Figures and Tables

**Table 1:** The lift-off area ($A_{0}$, expressed in ${Å}^{2}$/molecule), the collapse pressure ($\pi_{c},$ $\pi_{c1}$, $\pi_{c2}$, expressed in mN/m), and the collapse area (${A_{c},\;A}_{c1},\;A_{c2}$ expressed in ${Å}^{2}$/molecule) values obtained for the monolayers composed of phospholipids isolated from *L. gormanii* bacteria cultured on the non-supplemented (PL−choline) or choline-supplemented (PL+choline) medium, in the presence or absence of the LL-37 peptide at 20 °C or 37 °C.

	PL−choline (20 °C)	PL−choline + LL-37 (20 °C)	PL−choline (37 °C)	PL−choline + LL-37 (37 °C)	PL+choline (20 °C)	PL+choline + LL-37 (20 °C)	PL+choline (37 °C)	PL+choline + LL-37 (37 °C)
$A_{0}$	99.0	146.0	108.0	162.0	71.0	121.0	76.0	160.0
$\pi_{c}$	48.6	-	46.2	-	48.2	-	46.0	-
$A_{c}$	44.0	-	50.0	-	35.0	-	39.0	-
$\pi_{c1}/{\;\pi }_{c2}$	-	31.4/42.3	-	29.3/39.3	-	32.2/42.3	-	28.9/39.5
$A_{c1}/A_{c2}$	-	94.0/82.0	-	110.0/93.0	-	62.0/50.0	-	92.0/69.0

**Table 2:** Maximal ($C_{S,max1}^{-1}$, $C_{S,max2}^{-1}$, expressed in mN/m) and minimal ($C_{S,min}^{-1}$, expressed in mN/m) compression modulus values along with the corresponding surface pressure values ($\pi_{max1}$, $\pi_{max2}$, $\pi_{min}$, expressed in mN/m) for all obtained monolayers.

	PL−choline (20 °C)	PL−choline + LL-37 (20 °C)	PL−choline (37 °C)	PL−choline + LL-37 (37 °C)	PL+choline (20 °C)	PL+choline + LL-37 (20 °C)	PL+choline (37 °C)	PL+choline + LL-37 (37 °C)
$C_{S,max1}^{-1}$	107.0	62.0	109.0	69.0	119.0	74.0	116.0	79.0
$\pi_{max1}$	39.7	24.0	38.2	23.5	36.7	22.9	33.0	23.0
$C_{S,min}^{-1}$	-	34.0	-	22.0	-	44.0	-	22.0
$\pi_{min}$	-	31.4	-	29.3	-	32.2	-	28.9
$C_{S,max2}^{-1}$	-	48.0	-	40.0	-	62.0	-	48.0
$\pi_{max2}$	-	37.8	-	36.3	-	38.3	-	34.0

**Table 3:** The relative area change (∆A/A, expressed in %) for the PL−choline and PL+choline monolayers at π = 30 mN/m caused by the addition of LL-37 peptide into the subphase at 20 °C or 37 °C. The values given below correspond to the peptide effect at about 3 h after the injection.

	PL−choline (20 °C)	PL−choline (37 °C)	PL+choline (20 °C)	PL+choline (37 °C)
∆A/A	25	56	18	−70

## Data Availability

Not applicable.

## Supplementary Materials

The following supporting information can be downloaded at https://www.mdpi.com/article/10.3390/ijms241512039/s1.

## References

[1] Ephros M., Engelhard D., Maayan S., Bercovier H., Avital A., Yatsiv I. (1989). *Legionella gormanii* pneumonia in a child with chronic granulomatous disease. Pediatr. Infect. Dis. J..

[2] Han X.Y., Ihegword A., Evans S.E., Zhang J., Li L., Cao H., Tarrand J.J., El-Kweifi O. (2015). Microbiological and clinical studies of legionellosis in 33 patients with cancer. J. Clin. Microbiol..

[3] Lei C., Zhou X., Ding S., Xu Y., Yang B., Guo W., Song M., Yang M., Jia Y., Luo H. (2022). Case report: Community-acquired *Legionella gormanii* pneumonia in an immunocompetent patient detected by metagenomic next-generation sequencing. Front. Med..

[4] Svetlicic E., Jaén-Luchoro D., Klobucar R.S., Jers C., Kazazic S., Franjevic D., Klobucar G., Shelton B.G., Mijakovic I. (2023). Genomic characterization and assessment of pathogenic potential of *Legionella* spp. Isolates from environmental monitoring. Front. Microbiol..

[5] Qin T., Zhou H., Ren H., Liu W. (2017). Distribution of secretion systems in the genus *Legionella* and its correlation with pathogenicity. Front. Microbiol..

[6] Chmiel E., Galuska C.E., Koper P., Kowalczyk B., Urbanik-Sypniewska T., Palusińska-Szysz M., Fuchs B. (2022). Unusual lipid components of *Legionella gormanii* membranes. Metabolites.

[7] Palusińska-Szysz M., Szuster-Ciesielska A., Janczarek M., Wdowiak-Wróbel S., Schiller J., Reszczyńska E., Gruszecki W.I., Fuchs B. (2019). Genetic diversity of *Legionella pcs* and *PmtA* genes and the effect of utilization of choline by *Legionella* spp. on induction of proinflammatory cytokines. Pathog. Dis..

[8] Pastuszak K., Chmiel E., Kowalczyk B., Tarasiuk J., Jurak M., Palusińska-Szysz M. (2023). Physicochemical characteristics of model membranes composed of *Legionella gormanii* lipids. Membranes.

[9] Scharf S., Vardarova K., Lang F., Schmeck B., Opitz B., Flieger A., Heuner K., Hippenstiel S., Suttorp N., N’Guessan P.D. (2010). *Legionella pneumophila* induces human beta defensin-3 in pulmonary cells. Respir. Res..

[10] Yang B., Good D., Mosaiab T., Liu W., Ni G., Kaur J., Liu X., Jessop C., Yang L., Fadhil R. (2020). Significance of LL-37 on immunomodulation and disease outcome. BioMed Res. Int..

[11] Zeth K., Sancho-Vaello E. (2021). Structural plasticity of LL-37 indicates elaborate functional adaptation mechanisms to bacterial target structures. Int. J. Mol. Sci..

[12] Ridyard K.E., Overhage J. (2021). The potential of human peptide LL-37 as an antimicrobial and anti-biofilm agent. Antibiotics.

[13] Davies J.T., Rideal E.K. (1963). Interfacial Phenomena.

[14] Jurak M., Szafran K., Cea P., Martín S. (2021). Analysis of molecular interactions between components in phospholipid-immunosuppressant-antioxidant mixed Langmuir films. Langmuir.

[15] Kościuczuk E.M., Lisowski P., Jarczak J., Strzałkowska N., Jóźwik A., Horbańczuk J., Krzyżewski J., Zwierzchowski L., Bagnicka E. (2012). Cathelicidins: Family of antimicrobial peptides. A review. Mol. Biol. Rep..

[16] Neville F., Cahuzac M., Konovalov O., Ishitsuka Y., Lee K.Y.C., Kuzmenko I., Kale G.M., Gidalevitz D. (2006). Lipid headgroup discrimination by antimicrobial peptide LL-37: Insight into mechanism of action. Biophys. J..

[17] Sevcsik E., Pabst G., Richter W., Danner S., Amenitsch H., Lohner K. (2008). Interaction of LL-37 with model membrane systems of different complexity: Influence of the lipid matrix. Biophys. J..

[18] Dynarowicz-Łątka P., Hąc-Wydro K. (2004). Interactions between phosphatidylcholines and cholesterol in monolayers at the air/water interface. Colloids Surf. B.

[19] Jurak M., Mroczka R., Łopucki R. (2013). Properties of artificial phospholipid membranes containing lauryl gallate or cholesterol. J. Membr. Biol..

[20] Vogel V., Möbius D. (1988). Local surface potentials and electric dipole moments of lipid monolayers: Contributions of the water/lipid and the lipid/air interfaces. J. Colloid Interface Sci..

[21] Chachaj-Brekiesz A., Kobierski J., Wnętrzak A., Dynarowicz-Latka P. (2021). Electrical properties of membrane phospholipids in Langmuir monolayers. Membranes.

[22] Vanounou S., Parola A.H., Fishov I. (2003). Phosphatidylethanolamine and phosphatidylglycerol are segregated into different domains in bacterial membrane. A study with pyrene-labelled phospholipids. Mol. Microbiol..

[23] Epand R.M., Epand R.F. (2009). Domains in bacterial membranes and the action of antimicrobial agents. Mol. BioSyst..

[24] Sood R., Domanov Y., Pietiäinen M., Kontinen V.P., Kinnunen P.K.J. (2008). Binding of LL-37 to model biomembranes: Insight into target vs host cell recognition. Biochim. Biophys. Acta.

[25] Majewska M., Zamlynny V., Pieta I.S., Nowakowski R., Pieta P. (2021). Interaction of LL-37 human cathelicidin peptide with a model microbial-like lipid membrane. Bioelectrochemistry.

[26] Maget-Dana R. (1999). The monolayer technique: A potent tool for studying the interfacial properties of antimicrobial and membrane-lytic peptides and their interactions with lipid membranes. Biochim. Biophys. Acta.

[27] Crosio M.A., Via M.A., Camara C.I., Mangiarotti A., Pópolo M.G.D., Wilke N. (2019). Interaction of polyarginine peptide with membranes of different mechanical properties. Biomolecules.

[28] Rojewska M., Smułek W., Kaczorek E., Prochaska K. (2021). Langmuir monolayer techniques for the investigation of model bacterial membranes and antibiotic biodegradation mechanisms. Membranes.

[29] Bender J., Rydzewski K., Broich M., Schunder E., Heuner K., Flieger A. (2009). Phospholipase PlaB of *Legionella pneumophila* represents a novel lipase family: Protein residues essential for lipolytic activity, substrate specificity, and hemolysis. J. Biol. Chem..

[30] Palusińska-Szysz M., Jurak M., Gisch N., Waldow F., Zehethofer N., Nehls C., Schwudke D., Koper P., Mazur A. (2022). The human LL-37 peptide exerts antimicrobial activity against *Legionella micdadei* interacting with membrane phospholipids. Biochim. Biophys. Acta Mol. Cell Biol. Lipids.

[31] Ducret A., Chabalier M., Dukan S. (2014). Characterization and resuscitation of ‘non-culturable’ cells of *Legionella pneumophila*. BMC Microbiol..

[32] Barzyk W., Rogalska E., Więcław-Czapla K. (2013). Penetration of milk-derived antimicrobial peptides into phospholipid monolayers as model biomembranes. Biochem. Res. Int..

[33] Oren Z., Lerman J.C., Gudmundsson G.H., Agerberth B., Shai Y. (1999). Structure and organization of the human antimicrobial peptide LL-37 in phospholipid membranes: Relevance to the molecular basis for its non-cell-selective activity. Biochem. J..

[34] Lu F., Zhu Y., Zhang G., Liu Z. (2022). Renovation as innovation: Repurposing human antibacterial peptide LL-37 for cancer therapy. Front. Pharmacol..

[35] Henzler-Wildman K.A., Martinez G.V., Brown M.F., Ramamoorthy A. (2004). Perturbation of the hydrophobic core of lipid bilayers by the human antimicrobial peptide LL-37. Biochemistry.

[36] Martynowicz M.W., Rice A., Andreev K., Nobre T.M., Kuzmenko I., Wereszczynski J., Gidalevitz D. (2019). Salmonella membrane structural remodeling increases resistance to antimicrobial peptide LL-37. ACS Infect. Dis..

[37] Sevcsik E., Pabst G., Jilek A., Lohner K. (2007). How lipids influence the mode of action of membrane-active peptides. Biochim. Biophys. Acta.

[38] Takahashi H., Caputo G.A., Vemperala S., Kuroda K. (2017). Synthetic random copolymers as a molecular platform to mimic host-defense antimicrobial peptides. Bioconjug. Chem..

[39] Pastuszak K., Kowalczyk B., Tarasiuk J., Jurak M., Palusińska-Szysz M. (2023). Influence of the antimicrobial LL-37 peptide on *Legionella dumoffii* phospholipids adsorbed at the air–liquid interface. Sustainability.

[40] Bligh E.G., Dyer J.W. (1959). A rapid method of total lipid extraction and purification. Can. J. Biochem. Physiol..

[41] Amemura-Maekawa J., Hayakawa Y., Sugie H., Moribayashi A., Kura F., Chang B., Wada A., Watanabe H. (2004). Legioliulin, a new isocoumarin compound responsible for blue-white autofluorescence in *Legionella (Fluoribacter) dumoffi* under long-wavelength UV light. Biochem. Biophys. Res. Commun..

