# Supplementary material for: Insight into the Mechanism of Interactions between the LL-37 Peptide and Model Membranes of Legionella gormanii Bacteria
Source: Int J Mol Sci. 2023 Jul 27;24(15):12039. doi: 10.3390/ijms241512039 (PMC10418352; doi:10.3390/ijms241512039)
Supplement: Supplementary file 1 [file ijms-24-12039-s001.zip › ijms-2504729-supplementary.pdf]

International Journal of *Molecular Sciences*

MDPI

Supplementary Data

Insight into the Mechanism of Interactions between the LL-37 Peptide and Model Membranes of *Legionella gormanii* Bacteria

Katarzyna Pastuszak [1], Bozena Kowalczyk [2], Jacek Tarasiuk [2], Rafal Luchowski [3], Wieslaw I. Gruszecki [3], Małgorzata Jurak [1,*] and Marta Palusinska-Szysz [2]

[1] Department of Interfacial Phenomena, Institute of Chemical Sciences, Faculty of Chemistry, Maria Curie-Skłodowska University, Maria Curie-Skłodowska Sq. 3, 20-031 Lublin, Poland; katarzyna.pastuszak@mail.umcs.pl

[2] Department of Genetics and Microbiology, Institute of Biological Sciences, Faculty of Biology and Biotechnology, Maria Curie-Skłodowska University, Akademicka 19, 20-033 Lublin, Poland; b.kowalczyk746@wp.pl (B.K.); jacek.tarasiuk@mail.umcs.pl (J.T.); marta.palusinska-szysz@mail.umcs.pl (M.P.-S.)

[3] Department of Biophysics, Institute of Physics, Faculty of Mathematics, Physics and Informatics, Maria Curie-Skłodowska University, Radziszewskiego 10, 20-031 Lublin, Poland; rafal.luchowski@mail.umcs.pl (R.L.); wieslaw.gruszecki@mail.umcs.pl (W.I.G.)

* Correspondence: malgorzata.jurak@mail.umcs.pl

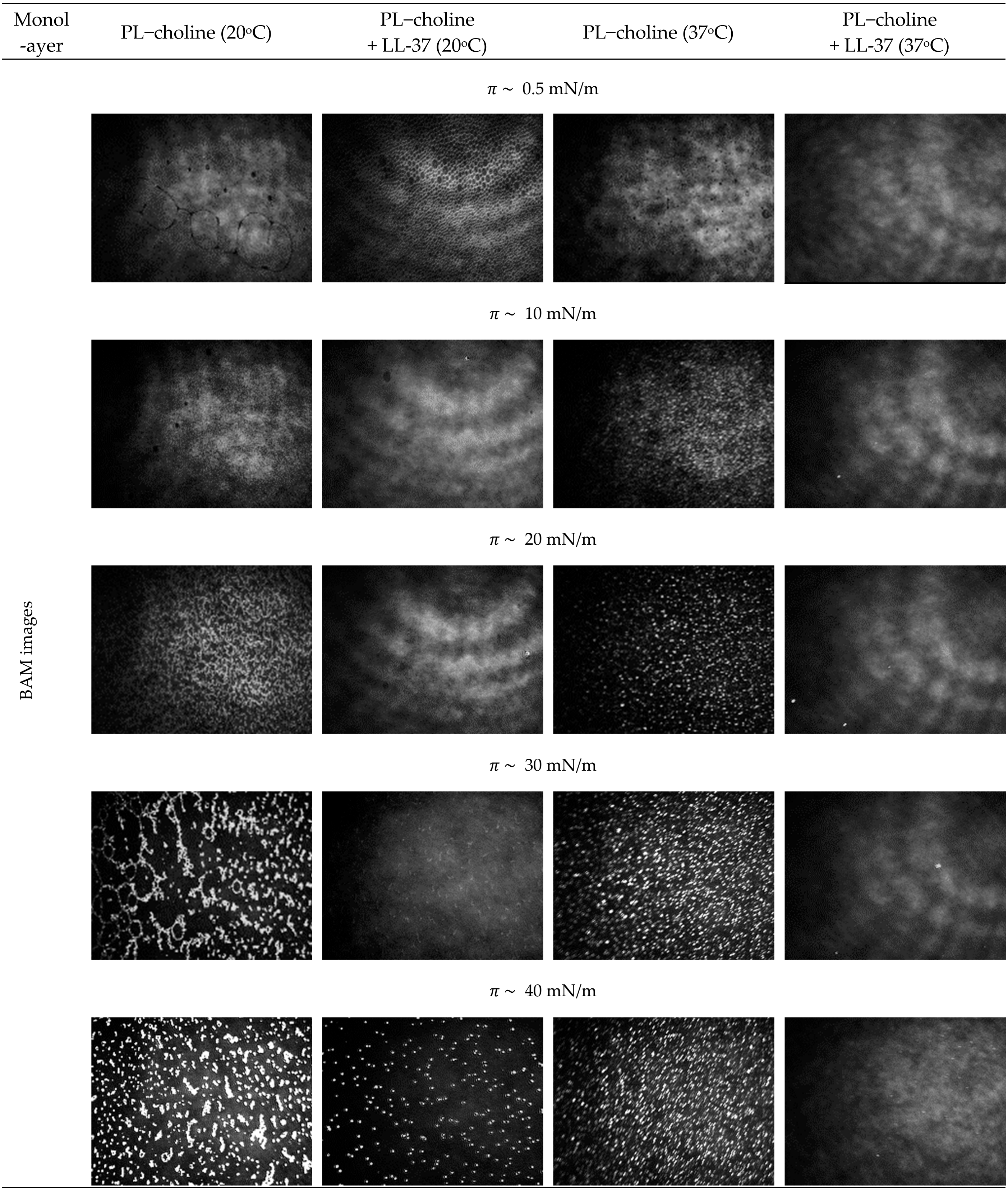

Figure S1. BAM images (720 μm × 400 μm) taken for the monolayers composed of phospholipids isolated from the *L. gormanii* bacteria cultured on the non-supplemented choline (PL−choline) medium, in the presence or absence of the LL-37 peptide at 20℃ or 37℃.

Monol-ayer	PL+choline (20°C)	PL+choline + LL-37 (20°C)	PL+choline (37°C)	PL+choline + LL-37 (37°C)

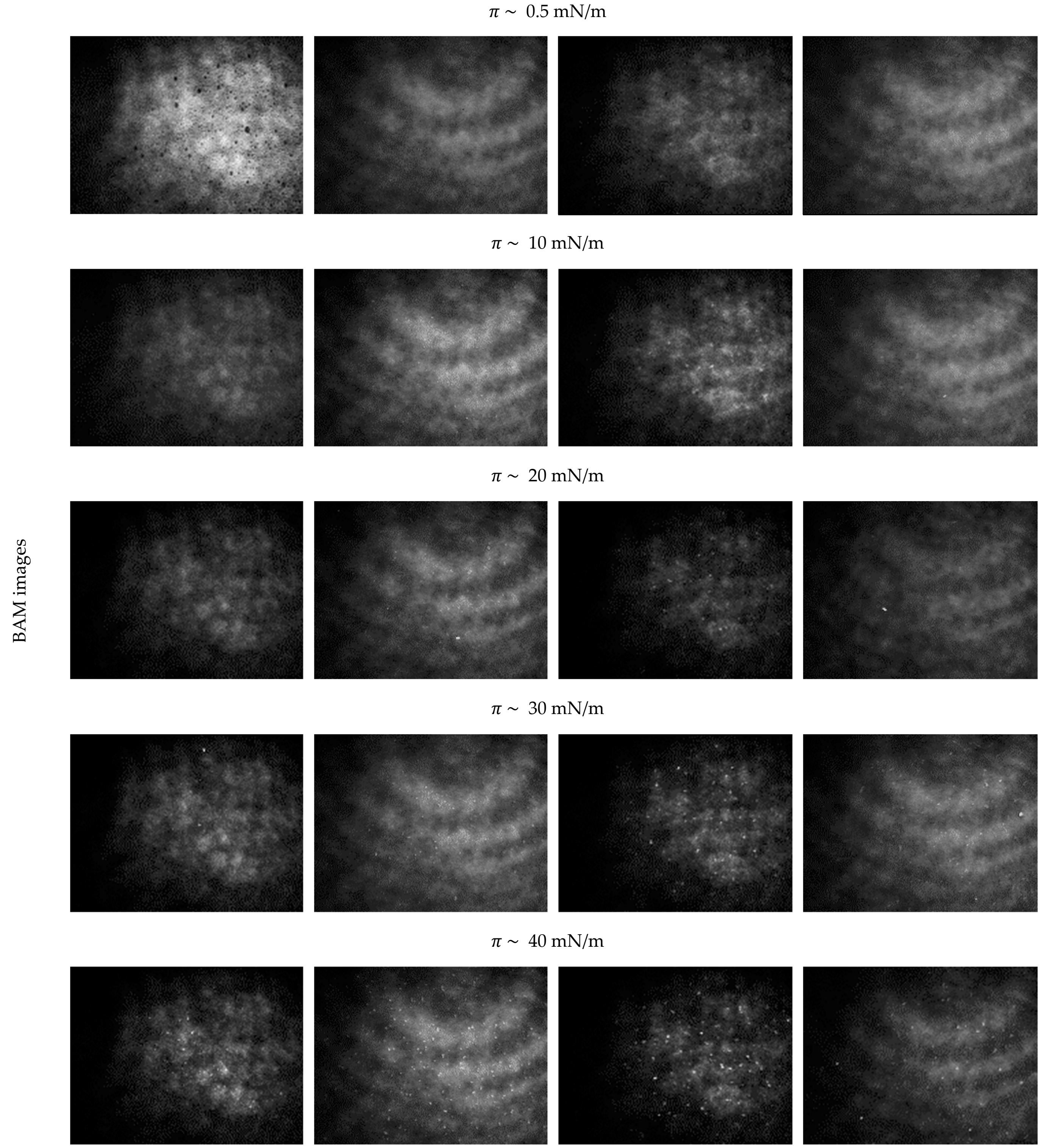

Figure S2. BAM images (720 μm × 400 μm) taken for the monolayers composed of phospholipids isolated from the *L. gormanii* bacteria cultured on the choline supplemented (PL+choline) medium, in the presence or absence of the LL-37 peptide at 20℃ or 37℃.
